# Whole-genome sequencing resolves a polyclonal outbreak by extended-spectrum beta-lactam and carbapenem-resistant *Klebsiella pneumoniae* in a Portuguese tertiary-care hospital

**DOI:** 10.1099/mgen.0.000349

**Published:** 2020-04-01

**Authors:** João Perdigão, Ana Modesto, A. L. Pereira, O. Neto, V. Matos, A. Godinho, Jody Phelan, James Charleston, Anton Spadar, Paola Florez de Sessions, Martin Hibberd, Susana Campino, A. Costa, F. Fernandes, F. Ferreira, A. B. Correia, Luisa Gonçalves, Taane G. Clark, Aida Duarte

**Affiliations:** ^1^​ Research Institute for Medicines (iMed.ULisboa), Faculty of Pharmacy, Universidade de Lisboa, Portugal; ^2^​ Clinical Pathology Unit. Hospital SAMS, Lisboa, Portugal; ^3^​ Infection Control Commission, Hospital SAMS, Lisboa, Portugal; ^4^​ Faculty of Infectious and Tropical Diseases, London School of Hygiene and Tropical Medicine, London, UK; ^5^​ Genome Institute Singapore, Singapore; ^6^​ Intensive Care Medicine Unit, Hospital SAMS, Lisboa, Portugal; ^7^​ Faculty of Epidemiology and Population Health, London School of Hygiene and Tropical Medicine, London, UK; ^8^​ Faculty of Pharmacy, Universidade de Lisboa, Portugal; ^9^​ Centro de Investigação Interdisciplinar Egas Moniz, Instituto Universitário Egas Moniz, Portugal

**Keywords:** ESBL, KPC, CTX-M, Gram-negative, Portugal, Lisbon

## Abstract

*
Klebsiella pneumoniae* has emerged as an important nosocomial pathogen, with whole-genome sequencing (WGS) significantly improving our ability to characterize associated outbreaks. Our study sought to perform a genome-wide analysis of multiclonal *K. pneumoniae* isolates (*n*=39; 23 patients) producing extended spectrum beta-lactamases and/or carbapenemases sourced between 2011 and 2016 in a Portuguese tertiary-care hospital. All isolates showed resistance to third-generation cephalosporins and six isolates (five patients) were also carbapenem resistant. Genome-wide-based phylogenetic analysis revealed a topology representing ongoing dissemination of three main sequence-type (ST) clades (ST15, ST147 and ST307) and transmission across different wards, compatible with missing links that can take the form of undetected colonized patients. Two carbapenemase-coding genes were detected: *bla_KPC-3_*, located on a *Tn4401d* transposon, and *bla_GES-5_* on a novel class 3 integron. Additionally, four genes coding for ESBLs (*bla_BEL-1_*, *bla_CTX-M-8_*, *bla_CTX-M-15_* and *bla_CTX-M-32_*) were also detected. ESBL horizontal dissemination across five clades is highlighted by the similar genetic environments of *bla_CTX-M-15_* gene upstream of IS*Ecp1* on a *Tn3*-like transposon. Overall, this study provides a high-resolution genome-wide perspective on the epidemiology of ESBL and carbapenemase-producing *K. pneumoniae* in a healthcare setting while contributing for the adoption of appropriate intervention and prevention strategies.

## Data Summary

All sequencing reads generated in this study have been deposited in the European Nucleotide Archive (ENA) (https://www.ebi.ac.uk/ena) under project PRJEB35084.

**Outcome**

Over the last decades *Klebsiella pneumoniae* emerged as an important healthcare-associated pathogen. To date, the present study comprises the largest genome-wide-based analysis on the epidemiology *K. pneumoniae* producing extended-spectrum beta-lactamases (ESBLs) and carbapenemases in Portugal. The study included 39 isolates from 23 patients infected and/or colonized with *K. pneumoniae* resistant to third-generation cephalosporins, five of which harboured carbapenemase-producing isolates, isolated in a Lisbon tertiary-care hospital between 2011–2016. This increasing incidence of ESBL and/or carbapenemase-producing *K. pneumoniae* is herein shown to consist in a multiclonal outbreak across distinct hospital wards mostly underpinned by three main clades: ST15, ST147 and ST307. This analysis is corroborated by the extremely narrow intra-clade pairwise core SNP distances (mean: 10.1 SNPs). The dissemination pattern is shown to be compatible with missing links in the transmission chains, which can take the form of colonized patients, healthcare personnel or other environmental niches that can effectively act as undetected reservoirs. Herein, WGS provided a fully resolved scenario when compared with classical multilocus sequence typing (MLST), enabling the segregation of unrelated isolates within the ST307 clade, which would otherwise be unresolved. Also, this approach contributes with precise genetic distances observed between isolates obtained from the same patient or within the same transmission chains, which informs on the identification and delineation of transmission clusters. Concerning the molecular basis of drug resistance, two carbapenemase-coding genes were found: *bla_KPC-3_*, located on a *Tn4401d* transposon, and *bla_GES-5_* on a novel class 3 integron. Together with the presence of the *bla_CTX-M-15_* gene upstream of IS*Ecp1* on a *Tn3*-like transposon across distinct clades, these findings reflect the ongoing lateral transfer of molecular determinants of resistance, with particular relevance to the dissemination of resistance to third-generation cephalosporins and carbapenems, which pose a serious challenge due to the limited therapeutic options. The data therefore supports the importance of screening measures to track colonization and, proper hygiene precautions while contributing to the adoption of appropriate intervention and prevention strategies.

## Introduction

*Klebsiella pneumoniae,* a common gut bacteria, causes problems when it moves outside the gut and causes infection with a disease spectrum ranging between urinary or respiratory tract infections to bloodstream infection [1]. *K. pneumoniae* has emerged as an important pathogen of public health importance, where the prevalence of multidrug-resistant forms has increased dramatically in recent years as an etiological agent of both community- and hospital-acquired infections. In the healthcare sector, *K. pneumoniae* is increasingly notorious as a frequent colonizer of hospitalized individuals from which it can easily disseminate and lead to nosocomial outbreaks [2, 3].

Antimicrobial-resistant (AMR) *K. pneumoniae* is prevalent in eastern and southern Europe, with Portugal experiencing an increasing prevalence of multidrug and carbapenem resistance among invasive isolates [4]. The main mechanisms of drug resistance underpinning clonal outbreaks of extended spectrum beta-lactamases (ESBL) and carbapenem resistance, are usually associated with *bla_TEM_-*, *bla_CTX-M_-* and *bla_KPC_*-producing strains [5–7]. However, beyond the usual clonal dissemination of AMR strains, resistance usually spreads through the capture of the resistance genes by mobile genetic elements such as integrons and transposons with intra- and cross-species mobilization usually potentiated by conjugative plasmids [8]. A wide diversity of distinct replicon types and plasmid scaffolds have been associated with the mobilization of drug resistance among several Enterobacteriaceae species in Portugal, namely IncFII and IncFIB plasmids [9]. Also, additional mobile genetic elements such as Tn*4401* transposons and class 1–3 integrons have been shown to promote the dissemination of drug resistance determinants [10–12].

Whole-genome sequencing (WGS) is revolutionizing genomic medicine and public health, and has significantly improved our ability to analyse outbreak scenarios, to characterize the transmission dynamics of important pathogens, and assess horizontal transfer mediated by resistance-mobilizing genetic elements [13]. This unprecedented resolution over classical genotypic methods such as pulse field gel electrophoresis and multilocus sequence typing (MLST) becomes particularly relevant to study and assess the nosocomial spread of AMR pathogens and more accurately evaluate infection control within healthcare institutions [14]. For example, WGS contributed to resolve a polyclonal outbreak scenario involving CTX-M-15-producing *K. pneumoniae* in a neurorehabilitation centre in Germany and, its real-time implementation in outbreak investigation involving *K. pneumoniae* carbapenemase (KPC)-producing Entrobacteriaceae in Australia has brought the ability to re-interpret a *prima facie* widespread outbreak down to distinct nosocomial transmission networks [15, 16]. To efficiently control and prevent the dissemination of AMR pathogens in hospital settings, the ability of early detection and the tracking of infected or colonized patients are crucial. In this context, undetected colonization upon patient admittance can be a major driver for the dissemination of AMR strains and the associated genetic elements underpinning cross-strain, and even cross-species, mobilization across healthcare institutions [17].

Here, we describe the first genome-wide analysis of a multiclonal *K. pneumoniae* outbreak in a Portuguese tertiary-care hospital along with the dissemination and lateral transfer of multiple ESBLs and carbapenemases encoded and mobilized by distinct genetic elements. The characterization of the outbreak isolates was performed using both traditional microbiology and molecular biology methods, as well as WGS, thereby enabling the comparison of isolates across different wards between 2011–2016.

## Methods

### Patients and bacterial isolates

At the beginning of 2011, an increase of ESBL-producing *K. pneumoniae* (ESBL-KP) was observed at the Intensive Care Unit of SAMS hospital. Since then, infection prevention and control measures have been established such as rectal and nasal swabs of patients upon entry to the hospital or into the Intensive Care Unit. Between 2013 and 2016, 22 patients were included in the study due to development of *K. pneumoniae* infection or colonization following hospital admission. Infection was defined by clinical and laboratory criteria, while colonization was defined by the absence of relevant clinical symptoms (Table 1). Thirty-seven isolates of *K. pneumoniae *-producing ESBL and/or carbapenemase, were isolated between 2013 to 2016. Two isolates, previously collected from rectal swab and blood culture from one patient at the Intensive Care Unit in January 2011, were also included in the study, for a total of 39 isolates and 23 patients.

### Microbiology methods

The biological samples collected were sent to the Microbiology Unit of the Hospital and were inoculated on specific culture medium for Gram-negative bacteria for detection of ESBLs or carbapenemases for rectal and nasal swabs, and additional Columbia agar with 5 % sheep blood plates for clinical specimens.

Identification and antibiotic susceptibility testing were performed using the VITEK Microbial Detection System (bioMérieux, La Balme-les-Grotte, France). European Committee on Antimicrobial Susceptibility Testing (EUCAST) guidelines were used for interpretation of antimicrobial susceptibility testing (2014) [18]. Phenotypic ESBL and KPC detection were performed both with the double-disc synergy test with ceftazidime +avibactam (Oxoid, UK) and ESBL +AMPC kit (Mastdiscs; Mast Group, UK) and KPC with boronic acid inhibition [19].

### Molecular microbiology methods

Total DNA was extracted from all strains using NZY Tissue gDNA Isolation kit according to the manufacturer’s instructions. PCR assays were performed to amplify the genes encoding for CTX-M-, KPC-, *aac(6´)-Ib-cr*, using primers and conditions previously described [6, 20, 21]. PCR and Sanger sequencing-based MLST was performed based on the sequence analysis of fragments of seven housekeeping genes: *rpoB* (beta-subunit of RNA polymerase), *gapA* (glyceraldehyde 3-phosphate dehydrogenase), *mdh* (malate dehydrogenase), *pgi* (phosphoglucoseisomerase), *phoE* (phosphorine E), *infB* (translation initiation factor 2) and *tonB* (periplasmic energy transducer). Details of the MLST scheme including amplification and sequencing primers, allele sequences and STs are available on the Institute Pasteur’s MLST website (https://bigsdb.pasteur.fr/klebsiella/klebsiella.html).

### WGS, genome assembly and annotation

All clinical isolates were subjected to WGS by preparing sequencing libraries from genomic DNA, previously extracted from cultures grown overnight in Mueller–Hinton agar, using the NZY Tissue gDNA Isolation kit (NZYTech, Portugal), as per the manufacturer’s recommendations. Indexed library preparation was performed using Covaris and the New England Biolabs (NEB) NEBNext Ultra DNA Library Prep Kit (NEB, MA, USA E7370) following the manufacturer’s recommended protocol. Sequencing was done on an Illumina HiSeq 4K high output using paired-end 2×151 bp reads.

Raw sequence reads were assembled using SPAdes v.3.8 as implemented and optimized in the Unicycler pipeline (https://github.com/rrwick/Unicycler) [22]. The best k-mer length determined for all assemblies was 127 bp with ranges for contig N50 between 124.8 and 362. 8 Kbp, contig count between 91 and 307, and largest contig between 294.7 and 819.8 kbp. The contigs were annotated using rast software [23].

### Phylogenetic analysis

For this purpose, high-quality SNPs were obtained by mapping raw sequenced reads to the reference genome of *K. pneumoniae* NTUH-K2044 (GenBank accession NC012731). Raw reads were trimmed for low-quality base calls using Trimmomatic v0.36 by cutting reads whose average quality falls below an average PHRED score of 20 in a 4 bp sliding window and retaining reads with minimum length of 36 bp. Filtered reads were mapped to the reference genome using the Burrows Wheeler Aligner tool (BWA-MEM algorithm) [24, 25]. The obtained alignments (BAM format) were deduplicated and reads around indels locally realigned using Picard Tools and GATK v.3.6 (IndelRealigner) [26]. Variant calling was performed using SAMtools/BCFtools and GATK (UnifiedGenotyper) software (MQ >23, DP >10) [27]. Only concordant variants were retained. An additional coverage-based step was carried out to validate and call additional alleles by assigning a missing call if the coverage depth did not reach a minimum of 20 reads or none of the nucleotides reached 75 % of the total coverage. SNP sites with an excess of 10 % missing calls were removed; none of the samples showed an excess of 10 % missing calls across the total number of SNP sites [28].

Only SNPs occurring at genome sites conserved across all samples were considered for downstream analysis and concatenated to a core SNP alignment. Also, SNPs were retained only for reference genome positions yielding 49/50 bp unique k-mers to remove SNP positions associated with low mappability regions. A total of 59 968 sites across 41 genomes were obtained for downstream phylogenetic analysis. Nucleotide substitution models were fitted to this dataset and ranked using R/Phangorn package and Akaike Information Criterium (AIC). The GTR nucleotide substitution model (- log likelihood: 511 129.2) was chosen as the best fit to the dataset. A maximum-likelihood phylogenetic tree was constructed using Seaview (PhyML) and tree topology statistically assessed using the approximate Likelihood Ratio Test (aLRT) [29, 30]. Isolate Kp4866 (* K. oxytoca *) recovered from the same setting was used to root the tree. The obtained phylogenetic tree was further annotated using Interactive Tree of Life (iTOL, https://itol.embl.de/) [31]. Additionally, a minimum-spanning tree (MST) was constructed and visualized using Phyloviz and the goeBURST algorithm therein implemented [32].

Additional publicly available sequence data for clinical isolates belonging to ST15, ST147 and ST307 were used as external outbreak controls to check the integrity of clades denoting local institutional outbreaks (Table S2) [15, 33–35]. These data underwent the same mapping and phylogenetic pipeline as described above.

### Clade dating analysis

beast was used to estimate the divergence dates across the ST15, ST147 and ST307 main phylogenetic clades herein detected using the date of isolation as tip dates. Initial runs of 50 million iterations were carried out for different permutations of tree priors and clock models and compared by marginal likelihood estimation using both stepping-stone and path sampling methods. Across all clades, a coalescent Bayesian skyline tree prior distribution under an uncorrelated relaxed lognormal molecular clock was found to best fit the data upon comparison by Bayes factor estimation. Using the latter tree prior and molecular clock along with a general time reversible substitution model, beast was run for 100 million iterations with sampling at each 1000 iterations and a burn-in of 10 million (10 %) iterations. Tracer was used to evaluate the convergence of multiple runs as well as the effective sample sizes (ESS). All statistics showed a value well above the minimum ESS threshold herein considered (ESS=100) with the analysis for all three clades (ST15, ST147 and ST307). Convergence was observed for all statistics over three independent runs under the same priors in each clade. LogCombiner was used to merge log and tree files from independent runs.

### *In silico* analysis of MLST, plasmid replicons, drug resistance associated genes and variants and capsular types

*In silico* MLST was performed using MLST 1.8 (https://cge.cbs.dtu.dk/). Plasmid replicons were detected using Abricate along with the PlasmidFinder database (https://cge.cbs.dtu.dk/). A minimum of 60 % coverage and 95 % identity (80 % for Col-like plasmids) were used as thresholds for plasmid replicon identification [36]. AMR genes were detected and identified using AMRFinder (https://ncbi.nlm.nih.gov/pathogens/antimicrobial-resistance/AMRFinder/) and the NCBI Bacterial Antimicrobial Resistance Reference Gene Database (accession PRJNA313047) using a 60 % coverage and 95 % identity thresholds. Capsular type (K-locus) and O-locus types were predicted using Kaptive Web (https://github.com/kelwyres/Kaptive-Web).

### Genetic environment of *bla_CTX-M-15_, bla_KPC-3_ and bla_GES-5_*

Contigs containing either *bla_CTX-M-15_*, *bla_KPC-3_* or *bla_GES-5_* were extracted from genome assemblies and aligned using mafft 7 software [37]. The genetic environments of all three genes were then reconstructed by incorporating annotation data and by blasting (nucleotide) full-length contigs, or the region comprising up to 10 Kbp upstream and downstream of the resistance gene, against the entire GenBank nucleotide collection. GFF3 files containing the annotation from the best hits obtained from the blast analysis were used to manually reannotate genetic features in these regions. Genetic comparison diagrams were generated by EasyFig software [38].

## Results

### Outbreak description and clinical isolates

A total of 22 patients with clinical history consistent with an ESBL/carbapenemase-producing *K. pneumoniae* infection and/or colonization already upon admittance were identified between 2013 and 2016 in SAMS Hospital, a tertiary-care hospital in Lisbon, Portugal. In 2011, six patients at the Intensive Care Unit were infected with a ESBL-producing *K. pneumoniae* ST15 strain [39], one of which was included in the present study for comparative purposes in a total of 23 patients. These patients were admitted and/or stayed at four distinct wards: Intensive Care Unit (*n*=11), Surgery (*n*=6), Medicine (*n*=4) and Observation Service (*n*=2). Eight patients (P1, P3, P4, P5, P7, P10, P19, P24) had a prior history of hospitalization at the same hospital in different wards or were transferred between wards during the hospitalization period. Five patients (P9, P11, P18, P25, P26) were referred from other hospitals in the region. The remaining ten patients (P2, P6, P8, P13, P14, P15, P16, P17, P21, P23) had no previous hospitalization history. According to the admittance timeline and rectal swab screening, eight patients (P1, P3, P4, P11, P18, P19, P25, P26) were colonized upon admission potentially due to previous hospitalization history and subsequently developed a healthcare-associated infection (HAI).

A total of 39 isolates were recovered for characterization by classical microbiology and molecular biology methods. Nineteen out of the 39 isolates were obtained from six distinct clinical specimens: urine (*n*=10), blood (*n*=3), respiratory tract secretions by double-lumen catheter (RTS/DLC; *n*=3); catheter (*n*=2) and intra-operative swab (IOSwab; *n*=1). The remaining 20 strains were identified from rectal swabs (Table 1). All 39 clinical isolates showed resistance to third-generation cephalosporins (*n*=39/39 to cefotaxime [CTX] and *n*=38/39 to ceftazidime [CAZ]). In all but two isolates, clavulanate failed to revert resistance to third-generation cephalosporins. The screening for AmpC-type cephalosporins by susceptibility testing to cefoxitin (FOX) revealed resistant isolates (*n*=27/39). Six isolates were carbapenem resistant (*n*=6/39 resistant isolates to imipenem [IPM]). Isolates were also resistant to fluoroquinolones (*n*=32/39) and aminoglycosides (*n*=27/39).

**Table 1.** Clinical features of patients infected/colonized by *Klebsiella pneumoniae* isolates recovered from clinical or surveillance cultures and respective characterization by MLST

Patient	Age (Gender)	Hospital ward	Date of hospitalization	Infection site (date of isolation)	Colonization site (date of isolation)	MLST	Observations
P1	78 (M)	ICU	24-10-2012		Rectal (O7-01-13)	ST15	Previously at Hospital SAMS
				RTS/DLC (14-01-13)		ST15	
					Perianal (14-01-13)	ST15	
				Urine (15-01-13)		ST15	
					Rectal (21-01-13)	ST15	
					Rectal (04-03-13)	ST15	
P2	91 (F)	Medicine	22-07-2013	Urine (24-07-13)		ST960	
P3	70 (M)	Observation Service	29-10-2013	Urine (29-10-13)		ST307	Previously at Hospital SAMS
		Surgery	05-09-2014	Urine (05-09-14)		ST307	
P4	81 (M)	ICU	07-12-2013	RTS/DLC (15-12-13)		ST37	Transfer from surgery ward to ICU at Hospital SAMS
P5	69 (M)	Surgery	18-02-2014		Rectal (18-02-14)	ST348	Previously at Hospital SAMS
P6	64 (M)	ICU	12-04-2014		Rectal (21-04-14)	ST15	
P7	69 (M)	Medicine	28-04-2014		Rectal (30-04-14)	ST423	Nursing home
							Previously at Hospital SAMS
P8	80 (M)	Surgery	08-05-2014	Urine (22-05-14)		ST423	
P9	61 (M)	ICU	14-09-2014		Rectal (16-09-14)	ST307	Previously at Hospital C
P10	71 (M)	ICU	23-08-2014		Rectal (17-09-14)	ST17	Transfer from Medicine to ICU at Hospital AMS
P11	54 (F)	Surgery	09-10-2014	Catheter (26-10-14)		ST348	Transfer from Hospital S
P13	77 (M)	Surgery	27-12-2014	Urine (21-01-15)		ST35	
P14	89 (F)	Surgery	12-03-2015	Urine (17-03-15)		ST70	
P15	51 (M)	ICU	08-04-2015	Urine (09-05-15)		ST15	
					Rectal (11-05-15)	ST15	
				RTS/DLC (07-06-15)		ST15	
P16	68 (M)	Medicine	27-04-2015	Blood (27-05-15)		ST307	Household
P17	76 (M)	ICU	26-05-2015		Rectal (29-05-15)	ST307	
				Catheter (20/06/15)		ST307	
				Blood (26/06/15)		ST307	
					Rectal (06-07-15)	ST307	
		Medicine	26-05-2015	Urine (02/08/15)		ST307	
P18	74 (M)	Medicine	28-05-2015		Rectal (01-06-15)	ST307	Admission at Hospital SAMS; Previously Hospital F
P19	66 (F)	ICU	01-07-2015		Rectal (07-07-15)	ST307	Admission ICU
				IOSwab (06-08-15)		ST307	Transfer from surgery ward to ICU at Hospital SAMS
					Rectal (17-08-15)	ST307	
P21	83 (F)	ICU	03-01-2016		Rectal (14-01-16)	ST147	
P23	35 (M)	ICU	14-03-2016		Rectal (21-03-16)	ST147	
				Urine (07-04-16)		ST147	
P24	80 (M)	Observation Service	26-03-2016		Rectal (26-03-16)	ST147	Nursing home; Previously at Hospital SAMS
P25	72 (F)	Observation Service	25-04-2016		Rectal (26-04-16)	ST147	Household; Previously Hospital C
P26	75 (M)	ICU	03-01-2011		Rectal (03-01-11)	ST15	Admission at ICU
				Blood (05-01-11)		ST15	Previously Hospital E

1- RTS/DLC – respiratory tract secretions by double-lumen catheter.

2- IOSwab – intra-operative swab.

### Genomic population structure and phylogenetic context

To ascertain the genomic population structure, we started by characterizing *in silico* the sequence type (ST) of each clinical isolate. Ten STs were detected: ST307 (6/23 patients; *n*=13/39 isolates), ST15 (4/23; *n*=12/39), ST147 (4/23; *n*=5/39), ST348 (2/23; *n*=2/39), ST423 (2/23; *n*=2/39), and ST70, ST35, ST37, ST960 and ST17 (all 1/23; *n*=1/39) (Table 2). Moreover, multiple isolates belonging to the same patients were consistently assigned to the same ST.

**Table 2.** Genetic features of all 39 multidrug-resistant *K. pneumoniae* isolates obtained from the 23 colonized/infected patients, organized by ST

ST	No. of patients	No. of strains	Drug resistance enzymes and mutations			Plasmid replicons	Capsular locus (KL) / antigen O (O)
			Carbapenemases	ESBLs	Other		
ST307	6	13	–	CTX-M-15	TEM-1, OXA-1, SHV-28, APH(3′′)-Ib, APH(6)-Id, AAC(3)-IIa*, AAC(6′)-Ib-cr, QnrB1, Dfr14, Sul2, Tet(a)*, CatB3 ParC[S80I], GyrA[S83I]	IncFIB(K), IncFIB(pQil)*, Col(MGD2)*, ColRNAI*	KL102/O2v2
ST15	4	12	–	CTX-M-15	TEM-1, OXA-1, SHV-28, APH(3′′)-Ib, APH(6)-Id, AAC(3)-IIa, AAC(6′)-Ib-cr5, DfrA14, Sul2, CatB3, ParC[S80I], GyrA[S83F]	IncFIB(K), IncFIB(pQil)*, IncFIA(HI1), IncFII, IncR, IncL/M*, ColRNAI*	KL112/O1v1
ST147	4	5	KPC-3, GES-5	BEL-1	TEM-1, OXA-9, SHV-11, APH(3′′)-Ib, APH(6)-Id, AadA1, AAC(6′)-Ib, DfrA14, Sul2, ParC[S80I], GyrA[S83I]	IncFIB(K), ColRNAI	KL64/O2v1
ST348	2	2	–	CTX-M-15	TEM-1, OXA-1, SHV-11, APH(3′′)-Ib, APH(6)-Id, AAC(3)-IIa, AAC(6′)-Ib-cr5, QnrB1, DfrA14, Sul2, Tet(a), CatB3,	IncFIB(K)	KL62/O1v1
ST423	2	2	–	CTX-M-32	SHV-11	IncFIB(K), IncN	KL8/O2v2
ST17	1	1	–	CTX-M-8	SHV-11, AadA2, DfrA21, Sul1, QacEdelta1,	IncFIB(K), IncFII, IncI1, ColRNAI	KL25/O5
ST35	1	1	–	CTX-M-32	SHV-33, Tet(D)	IncFIB(K)	KL22/O1v1
ST37	1	1	–	CTX-M-15	TEM-1, OXA-1, SHV-11, APH(3′′)-Ib, APH(6)-Id, AAC(3)-IIa, AAC(6′)-Ib-cr5, QnrB1, DfrA14, Sul2, Tet(a), CatB3	IncFIB(K), IncFIB(Mar)	KL15/O4
ST70	1	1	–	CTX-M-15	TEM-1, OXA-1, SHV-32, APH(3′′)-Ib, APH(6)-Id, AAC(3)-IIa, AAC(6′)-Ib-cr5, QnrB1 DfrA14, Sul2, CatB3	IncFIB(K)	KL136/O1v1
ST960	1	1	KPC-3	–	TEM-1, SHV-164, APH(3′′)-Ib, APH(6)-Id, DfrA14, Sul2	IncN, ColRNAI	KL10/O2v2

*Resistance genes or plasmid replicons highlighted with an asterisk (*) were not detected across all isolates within the same ST, see Table SX.

**Fig. 1.**
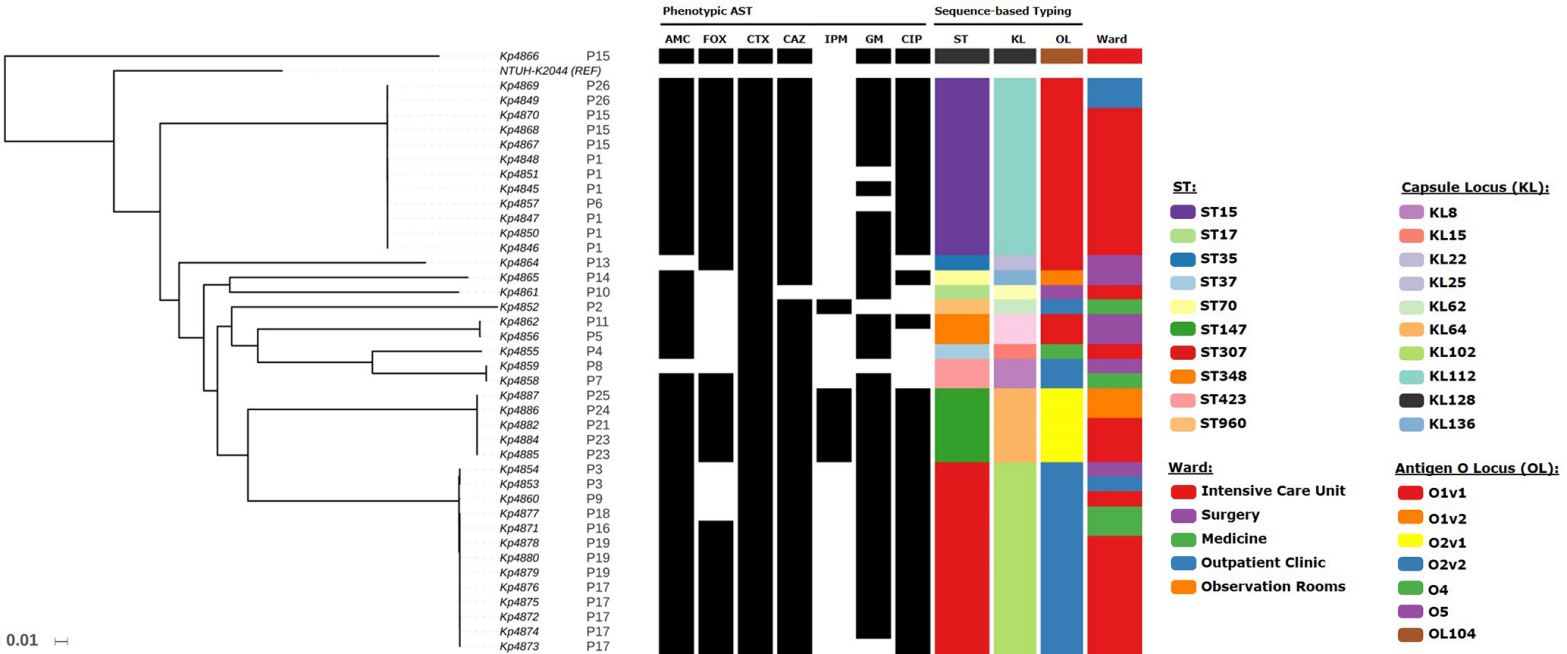
Maximum-likelihood phylogenetic tree based on 59 968 variant SNPs for the 40 clinical isolates included in the study. The tree was rooted with *Klebsiella oxytoca* Kp4866 sequenced in the study. The tree is shown as a phylogram (see Fig. S1 for the cladogram) and annotated with phenotypic drug susceptibility data to amoxicillin-clavulanate (AMC), cefoxitin (FOX), cefotaxime (CTX), ceftazidime (CAZ), imipenem (IPM), gentamicin (GM) and ciprofloxacin (CIP) where black rectangles indicate phenotypic resistance. Isolate ST, capsular locus type (KL), antigen O locus type (OL) and ward of isolation are also annotated on the tree according to the colour-coded legends.

A maximum-likelihood phylogenetic tree was generated based on 59 968 genome-wide core SNPs (Figs 1 and S1). The tree was topologically structured by ST and patient, with each forming monophyletic clades comprised by closely related clinical isolates. A single exception was strain Kp4857 (P6, ST15) that emerges within the group mostly formed by strains isolated from patient P1. The distribution of capsular locus (KL) types across the tree denoted specific associations with ST, where each KL type occurred only in one ST and was common to all isolates belonging to that ST (Fig. 1). The most prevalent KL types were KL64, KL112 and KL102 and linked to ST147, ST15 and ST307, respectively. Conversely, antigen O locus (OL) types were dispersed throughout the phylogenetic tree in a homoplasic fashion. In particular, OL types O1v1 were associated with ST15, ST35 and ST348 whereas O2v2 type was associated with ST307, ST423 and ST960.

A common trait across the three major clades (ST15, ST307 and ST147) is that most of the contributing patients were admitted to the intensive care unit with strains isolated during their stay within this ward. However, in ST147, two out of the four patients’ clinical isolates were detected upon admittance to the observation stay ward and showed an early and more basal branching within the ST147 clade. Also, in ST15 and ST307 clades, the basal branches include (but are not limited to in ST307) patients referred from an outpatient clinic.

### Resistome and plasmids

Analysis of the resistomic landscape revealed uniform patterns of resistance-conferring genes across isolates from the same patient and belonging to the same ST phylogenetic clade. An exception to this general observation concerns aminoglycosides resistance where two isolates (Kp4853, Kp4854) from patient P3 were negative for *aac(3)-IIa* gene, which was present in the remaining ST307 isolates (Table S1). ST70, ST307 and ST15 clades showed similar aminoglycoside-associated resistome patterns (Table 2). However, contrarily to the remaining ST307 isolates, isolate Kp4860 was negative for *tet(A*). Regarding resistance to fluoroquinolones, it was mainly associated with either chromosomal mutations on *parC* and *gyrA* genes or, plasmid mobilization of *qnrB1*. ST147, ST15 and ST307 showed a S80I missense mutation on ParC with concomitant GyrA S83I mutation among ST147 and ST307 strains or, the S83F mutation by ST15 isolates. No mutations were detected among the quinolone resistance-determining regions of the DNA gyrase and topoisomerase IV genes of isolates belonging to ST17, ST35, ST423 or ST960, which is consistent with susceptibility to ciprofloxacin. However, ST37 and one of the two ST348 isolates, also susceptible to ciprofloxacin, showed concomitant presence of AAC(6’)-Ib-cr5, and QnrB1 coding genes (Table 2). The same genotype was nevertheless found in a second ST348 isolate and on the single ST70 isolate, both resistant to ciprofloxacin.

A more diverse scenario was observed regarding beta-lactamases, with the presence of two carbapenemase-coding genes (*bla_KPC-3_* and *bla_GES-5_*), four genes coding for ESBLs (*bla_BEL-1_*, *bla_CTX-M-8_*, *bla_CTX-M-15_* and *bla_CTX-M-32_*) and nine genes coding for narrow/broad spectrum beta-lactamases (*bla_TEM-1_*, *bla_DHA-1_*, *bla_OXA-1_*, *bla_OXA-9_*, *bla_SHV-11_*, *bla_SHV-28_*, *bla_SHV-32_*, *bla_SHV-33_*, *bla_SHV-164_*). For the distribution of carbapenemase-coding genes, co-occurrence of a *bla_KPC-3_* and *bla_GES-5_* genes were observed for all ST147 isolates, whereas a KPC-3-coding gene was detected in the ST960 clinical isolate (Kp4852/P2). Interestingly, all ST307 and ST15 isolates displayed similar beta-lactamase gene patterns (*bla_TEM-1_*, *bla_OXA-1_*, *bla_SHV-28_* and *bla_CTX-M-15_*). This observation held for ST348/ST37 (*bla_TEM-1_*, *bla_OXA-1_*, *bla_SHV-11_* and *bla_CTX-M-15_*) (Table 2).

Concerning plasmid replicon types, a total of 11 replicon types were found: IncFIA(HI1) (*n*=12), IncFIB(K) (*n*=38), IncFIB(pQil) (*n*=15), IncFIB(Mar) (*n*=1), IncFII (*n*=13), IncI (*n*=1), IncL/M (*n*=1), IncN (*n*=3), IncR (*n*=13), Col(MGD2) (*n*=10) and ColRNAI (*n*=18). It was only possible to phase replicon type and AMR genes to the same contig on two occasions: (i) ST17/Kp4861 showed colocalization of Sul1, QacEDelta1, AadA2 and DfrA21 coding genes on a IncFII replicon; and (ii) ST147 isolates showed *bla_BEL-1_* and *bla_GES-5_* colocalization on a ColRNAI plasmid. A clear predominance of IncFIB(K) was observed across nine of the ten STs detected, and across all isolates within those types. This observation contrasts with IncFIB(pQil), IncL/M, Col(MGD2) and ColRNAI replicons, which appear to show variation within ST307 and ST15 isolates. This variability is in fact detectable at the patient level: ColRNAI, IncL/M and IncFIB(pQil) were not detected among all P1 isolates; IncFIB(pQil) and ColRNAI in P17 isolates; and, ColRNAI in P19 isolates. These data show that plasmid loss and lateral transfer is highly dynamic even at the patient level.

### Genetic context of *bla_CTX-M-15_*, *bla_GES-5_* and *bla_KPC-3_*

**Fig. 2.**
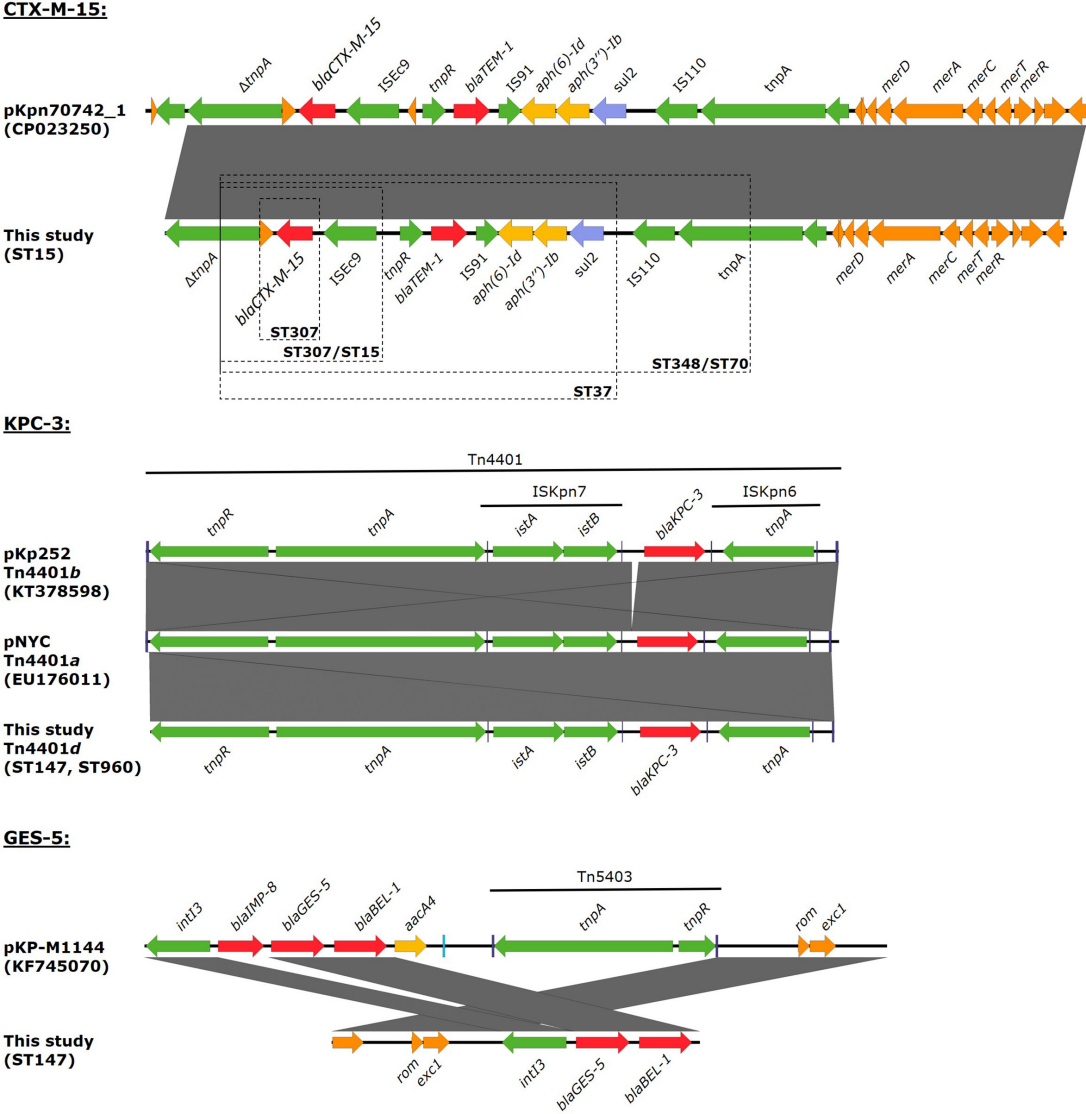
Genetic environment of CTX-M-15, KPC-3 and GES-5 found in this study and across multiple STs. Genes are highlighted according to its association with resistance to beta-lactams (red), aminoglycosides (yellow), sulfonamides (blue) or association with mobile elements or gene mobilization (green). Blue boxes highlight inverted repeats associated with mobile elements. Dashed boxes on the CTX-M-15 genetic context provides an approximate perspective on the reconstructed regions across different STs due to variable contig length across different STs and isolates.

The genetic environment of *bla_CTX-M-15_*-, *bla_GES-5_*- and *bla_KPC-3_*-coding genes was evaluated to infer any common origins between other published datasets. *bla_CTX-M-15_* was detected among ST15, ST307, ST37, ST348 and ST70 isolates. This gene was always located upstream of the insertion sequence IS*Ecp1*, which belongs to the IS*1380* family and is able to mobilize *bla_CTX-M_* genes by a transposition process [40]. The latter were found on a Tn3-like transposon structurally similar to the previously reported pEC_Bactec plasmid, of the IncI1 incompatibility group, from an *Escherichia coli* isolated from the joint of a horse [41]. This Tn3-like plasmid encompasses, along with a TEM-1, a partial *tnpA* ORF disrupted by IS*Ecp1-bla_CTX-M-15_*. Also, it is colocalized with (i) the *aph(6)-Id*, *aph(3’’)-Ib* and *sul2* genes, all of which upstream of an IS*91* element; and (ii) a narrow-spectrum *mer* operon located on a Tn*501* transposon, part of the Tn*3* transposon family [42]. Although the length of the contigs harbouring *bla_CTX-M-15_* varied between different isolates, the data suggests a common origin by lateral transfer across multiple STs (Fig. 2).

*bla_KPC-3_* was detected among ST147 and ST960 strains, and present in a similar genetic environment as part of the Tn*4401d* isoform, which is characterized by a 68 bp deletion between *istB* and KPC [43]. This transposon type is usually associated with *bla*_*KPC-2*_ although it has also been detected in KPC-3-producing isolates from northern Portugal [10]. KPC-3 has been previously associated with ST147/wzi64/K14.64 isolates but not with concomitant BEL-1/GES-5 [10]. Here, *bla_BEL-1_* and *bla_GES-5_* were located on a new class 3 integron In*4885* showing a similar structure to the In*1144* integron described from an IMP-8-producing ST252 *K. pneumoniae* isolate identified in a Portuguese hospital in 2009, although In*4885* lacks the IMP-8 gene cassette despite being also located on a ColRNAI plasmid [44].

### Phylodynamic transmission network

We sought to understand the transmission dynamics of the detected strains across the different hospital wards. Based on the core SNP alignment, within each ST, the average SNP distance was 10.1 SNPs (range: 2–77), with variation across ST clade (Table 3). SNP distances observed between isolates from different STs were greater than 9 245 SNPs and within patients, core SNP pairwise distance between isolates ranged between 2 and 15 SNPs (Table 3; Fig. S2)

**Table 3.** Node age estimates for the three main clades (ST15, ST147 and ST307) along with age estimates for patients with more than two *K. pneumoniae* isolates included in the study and monophyletic intra-clade branches including multiple patients. First isolation date and hospitalization data is provided for individual patients listed in the table and core SNP distance intervals are shown for each clade, patient or sub-branch. The difference between the node age estimate and hospitalization date expresses the difference, in days, between the phylogenetic-based estimate for coalescent patient nodes and the date of the patient admittance to the hospital

Clade/patient	Node age mean [95 % HPD interval]	Node age mean	First isolation date	Hospitalization date	Core SNP distance	Node age and hospitalization date difference (days)
**ST15**	2010.7650 [2010.2319,2011.0115]	6 October 2010	–	–	2–25	–
P1	2012.7817 [2012.3635,2013.0152]	12 October 2012	7 January 2013	24 October 2012	2–15	12
P15	2014.9512 [2014.1683,2015.3495]	13 December 2014	9 May 2015	8 April 2015	4–7	116
P26	2010.8521 [2010.4877,2011.0115]	7 November 2010	3 January 2011	3 January 2011	2	57
P1/P6	2012.7817 [2012.3635,2013.0152]	12 October 2012	–	–	3	–
						
**ST147**	2016.0163 [2015.9482,2016.0390]	5 January 2016	–	–	3–14	–
P23	2016.1953 [2016.1274,2016.2223]	11 March 2016	21 March 2016	14 March 2016	6	3
P24/P25	2016.1969 [2016.1022,2016.2360]	12 March 2016	–	–	8	–
P23/P24/P25	2016.1557 [2016.0201,2016.2216]	25 February 2016	–	–	4–8	–
						
**ST307**	2013.2348 [2012.2717,2013.8302]	26 March 2013	–	–	2–77	–
P17	2015.2910 [2015.0870,2015.4074]	16 April 2015	29 May 2015	26 May 2015	2–9	40
P19	2015.4190 [2015.2207,2015.5180]	1 June 2015	7 July 2015	1 July 2015	2–5	30
P3	2013.6829 [2013.3743,2013.8300]	6 September 2013	29 October 2013	29 October 2013	10	53
P16/P18	2015.3125 [2015.1282,2015.4051]	24 April 2015	–	–	6	–

**Fig. 3.**
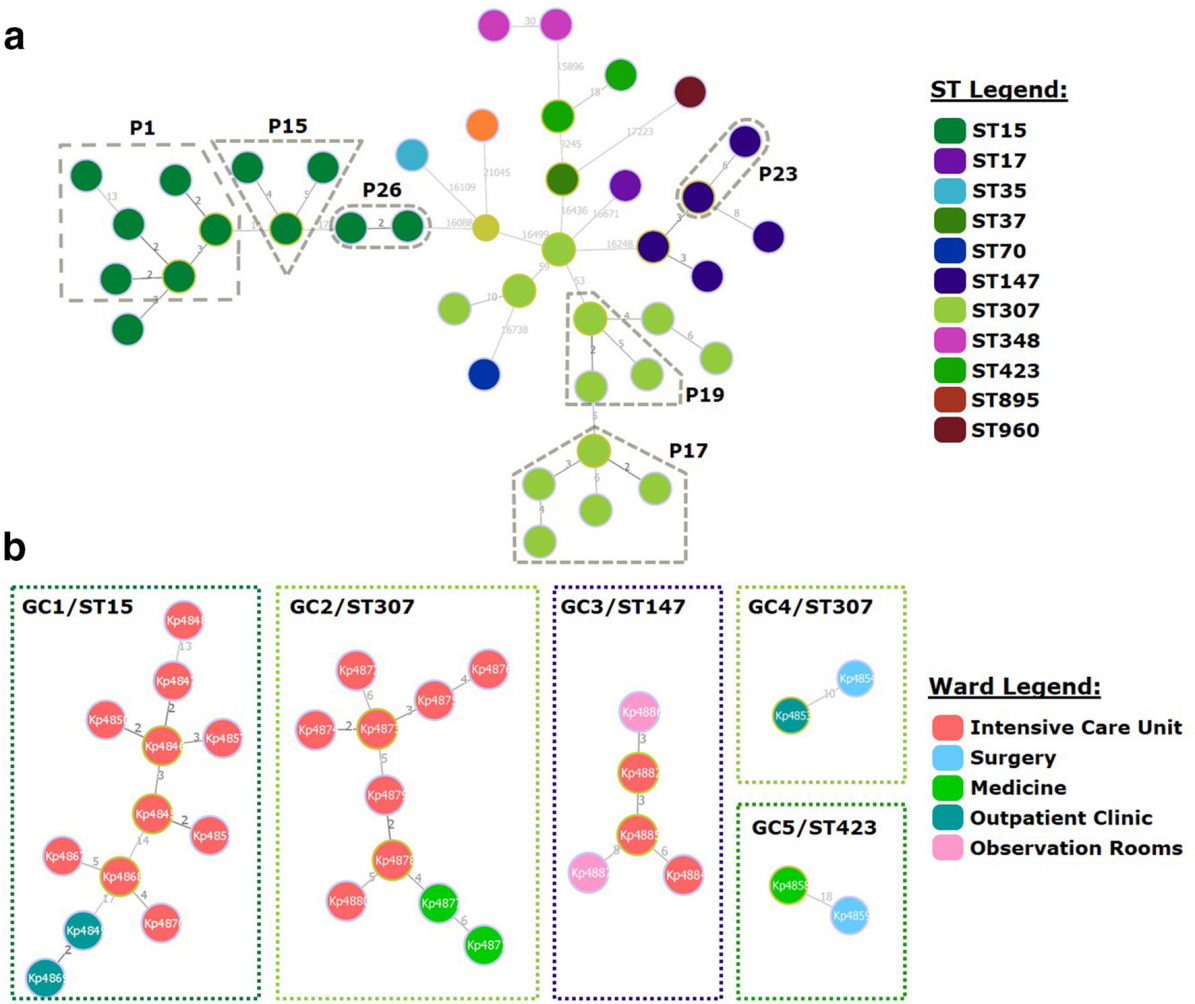
goeBURST full MST (a) of all 39 *K *. *pneumoniae* clinical isolates included in the study along with the *K. oxytoca* Kp4866 and genomic clusters (b, GC1-5) found by applying a cutoff distance of 23 core SNPs. In (a_, node colouring depicts the ST of each clinical isolate present at each node in the tree according to the ST colour legend. (b) shows all five genomic clusters detected in the study with node coloring representing the ward at which the isolate was detected therefore illustrating strain dissemination across multiple wards in all five clusters. Numbers at each branch indicate the distance between nodes in SNPs; links with shorter distances are coloured in black and longer distances in greyscale.

A minimum spanning tree was constructed for all clinical isolates and, using a SNP pair-wise distance cutoff of 23 SNPs to delineate genomic clusters with a high likelihood of epidemiological linkage [45], all strains belonging to the same ST could be grouped in the same genomic cluster (Fig. 3) except for ST307 and ST348 strains. ST307 strains included three isolates (Kp4853, Kp4854 and Kp5860) from two patients (P3 and P9) with SNP distances of 59 and 53 from the nearest ST307 node. Using external outbreak controls for the three main clades in the study, the new minimum spanning tree topology showed that isolates Kp4853 and Kp4854 (P3) grouped closer with *K. pneumoniae* strains sourced from the UK (Assembly Accession ASM216703v1) than with the remaining ST307 isolates from Portugal (Fig. S3). This observation suggests that patients P3 and P9 infection/colonization with *K. pneumoniae* is epidemiologically unrelated with each other and with the remaining ST307 strains. Similarly, ST348 isolates Kp4856 (P5) and Kp4862 (P11) separated by 30 SNPs were of questionable epidemiological relatedness. This analysis also stresses the importance of external controls to assess clade integrity in an outbreak investigation scenario, particularly in cases such as this where lack of additional epidemiological data also precludes us from resolving the different sources for these particular strains.

**Fig. 4.**
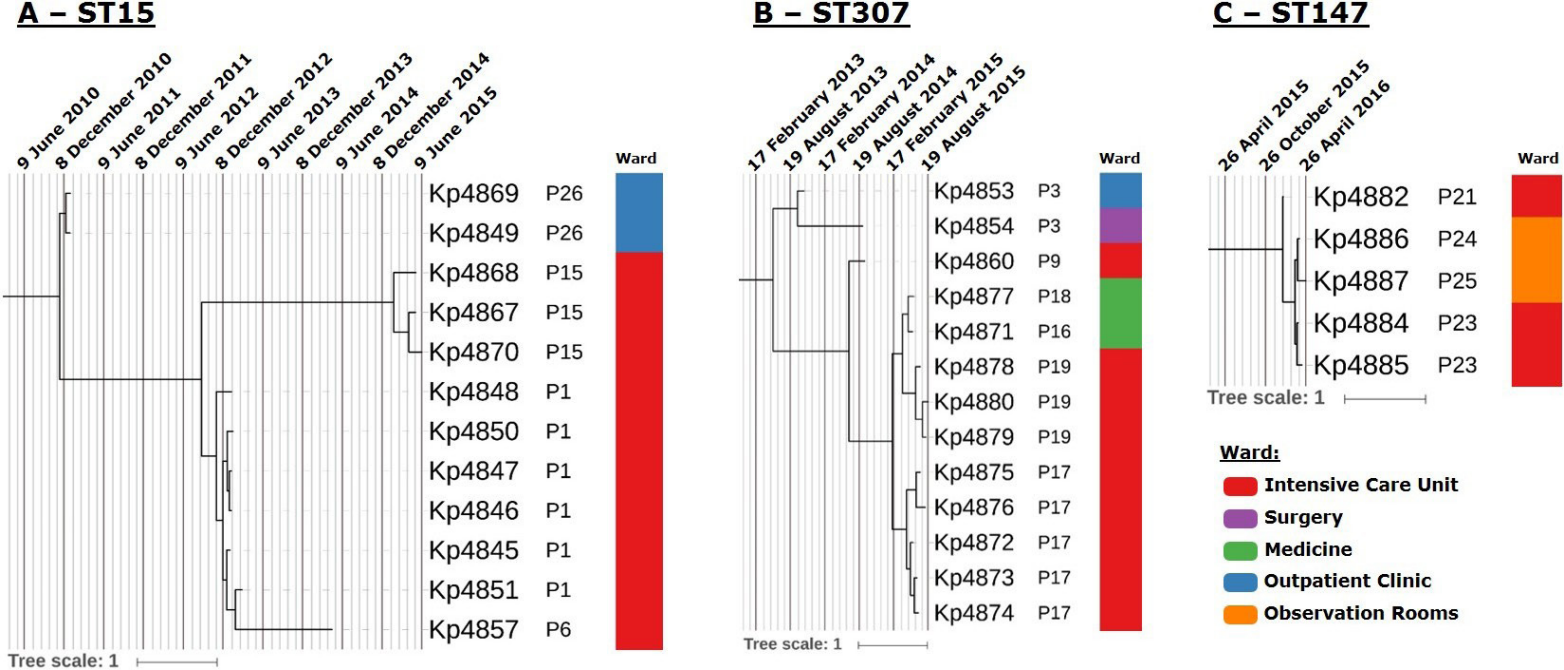
Maximum clade credibility time-scaled trees obtained for the ST15 (A), ST307 (B) and ST147 (C) strains by Bayesian phylogenetic reconstruction to estimate node divergence times across each clade. Trees are annotated with grid axis containing major and minor markings representing 0.5 and 0.1 years, respectively, and with patient ID code and ward of isolation annotated by a colour strip according to the legend provided.

Using the isolation date to time-scale the phylogenetic sub-tree of ST15, ST147 and ST307 strains allowed us to estimate when the most recent common ancestor (MRCA) of each clade split (Table 3). For ST15 and ST307, the MRCA of each clade is thought to have diverged on October 2010 (95 % highest posterior density [HPD]: March 2010–January 2011) and March 2013 (95 % HPD: April 2012–October 2013), respectively. ST147 MRCA is estimated to have diverged on January 2016 (95 % HPD: December 2015–January 2016), which is temporally closer to the detection of the first ST147 isolates in the study, and suggests more recent introduction and dissemination across the hospital setting. Dating the MRCA for patients with multiple available samples revealed that in most instances the estimated MRCA age is within 12–57 days of the patient’s admittance date. However, for patient P15 a difference of 116 days exists between the phylogenetic-based estimates and the admittance date suggesting a prior infection or colonization event (Table 3). Within the time-scaled phylogenetic trees, the ST15 and ST307 clades denote a similar pattern of initial detection of patient colonization/infection at the outpatient clinic followed by admittance to the hospital, ensuing dissemination through the intensive care unit and, in ST307, also to the medicine ward (Fig. 4). On the other hand, ST147 strains are thought to have initially spread within the intensive care unit followed by dissemination to the observation room ward in the first quarter of 2016. The data also show that the P6 clinical isolate diverges directly from P1 isolates, with the latter being detected approximately 1 year before. The coalescent MRCA for these strains is thought to have occurred in October 2012 (95 % HPD: May 2012–January 2012) (Table 3, Fig. 4).

## Discussion

The emergence of *K. pneumoniae* across European healthcare sectors currently poses a major concern on hospital infection control, namely when ESBL and carbapenemase-producing strains are involved. Over the past two decades, owing to the dissemination of CTX-M type β-lactamases, there have been numerous reports about the increasing incidence of Enterobacteriaceae-producing ESBLs. In this study, one of the primary working hypotheses was to verify if the ESBL-producing *K. pneumoniae* ST15 strain, which infected six intensive care unit patients in 2011 is related to the strains identified later in the years 2013–2016 [39]. Over this time period and using high-resolution genome-wide analysis of 39 *K *. *pneumoniae* isolates, the present study demonstrates the occurrence of sporadic multiclonal *K. pneumoniae* outbreaks along with the dissemination and lateral transfer of multiple ESBLs and carbapenemases mobilized by distinct genetic elements.

The data gathered by this study for this hospital institution highlights the growing problem of multidrug resistance, where 12 out of 19 patients with either ESBL or carbapenemase producing *K. pneumoniae* showed concomitant resistance to aminoglycosides and fluoroquinolones. This finding stresses the association between resistance to third-generation cephalosporins and carbapenems with increasing resistance to other antimicrobial drug classes, and a poor therapeutic outcome [46]. This association has been recognized by the European Centre for Disease Control.

The importance of colonization prior to the development of clinical disease is emphasized by the fact that five patients showed colonization with the same strain prior to the progression to a clinical stage. In two patients this progression evolved to complicated bloodstream infections. This data could be corroborated by WGS analysis of both isolates gathered from colonization samples, herein rectal swabs, and other biological products reflecting clinical disease where, all but one patient formed monophyletic clades with an intra-patient SNP-distance between isolates of up to 15 SNPs. A recent study by Sherry *et al.* [45] proposes that pairwise SNP distances between isolates of up to 23 SNPs suggests local transmission, which, in our study, is congruent with the intra-patient pairwise SNP distances but, importantly, also corroborates the conclusion that distinct ST307 transmission chains existed in this hospital. Beyond the high-resolution power offered by WGS we also stress the importance of including external outbreak control strains to better ascertain the epidemiological relatedness between isolates over the topological structure of a phylogenetic tree. Compared to classical MLST typing, WGS offers a fully resolved scenario that became particularly useful for ST307 strains which, presumably encompass epidemiologically unrelated patients that could otherwise be considered as part of the same local transmission chain based on MLST alone, particularly in the absence of good epidemiological data and adequate screening for carriage control and patient isolation protocols. In such situations, WGS provides a finer resolution to the investigation of the genomic relatedness between isolates with the potential to generate distinct insights on the transmission dynamics of these strains. Herein, the identification of a ST307 strain among patients P3 and P9 is contemporary but displaced in time from the remaining patients harbouring ST307 strains.

Remarkably, *K. pneumoniae* ST15 strains have been a persistent CTX-M-15-producing clone that was found in the Lisbon hospital since 2011, represented in this study by patient P26, and three other patients (P1; P6; P15) hospitalized in the years 2013, 2014 and 2015, respectively. The phylogeny of 12 *K *. *pneumoniae* ST15 isolates underscores a highly structured clonal population with the core genome alignment of these strains revealing distances of between 2 and 25 SNPs. Using the 23 SNP cutoff mentioned above, this data conveys the notion of ESBL-producing ST15 *K. pneumoniae* persistence and ongoing transmission in this hospital for at least 4 years [45]. By having ST15 isolates sampled across this period (2011–2015) it was possible to trace back, using Bayesian phylogenetic dating, the likely emergence of ST15 in this hospital to the end of 2010, a period compatible with the initial detection of ESBL-producing strains in this same setting.

The rapid occurrence of CTX-M-producing strains in Enterobacteriaceae has been documented by several longitudinal surveillance studies with its widespread distribution observed not only in humans but also in animals and in the environment. The emergence of these globally distributed strains is intimately associated with a conserved IncFIIK/IncFIBK ESBL plasmid harbouring the *bla_CTX-M-type_* and several other antibiotic multiresistant determinants [47]. Our results suggest that the emergence CTX-M-type might result from the horizontal transfer of an IncFIBK plasmid common in *K. pneumoniae* strains across different STs. In addition to this epidemic IncFIK plasmid, other genetic mobile elements were found as insertion sequences, including IS*Ecp1* located upstream of the *bla_CTX-M-15_* gene. IS*Ecp1* (synonym, ISEc9) harbouring an IS*1380* family transposase is one of the most important elements associated with CTX-M-types (UniProtKB: Q6SLK9). IS*Ecp1* is composed of an ORF encoding a transposase with 420 amino-acids and two imperfect and inverted repeats. IS*Ecp1* can mobilize the downstream-located *bla*_*CTX-M*_ gene and provide a promoter for its expression [48]. Also regarding ST15 strains, the P6 clinical isolates appear to be nested phylogenetically in the clade formed by P1 isolates, although P6 was admitted to the hospital approximately 1 year after P1. This is suggestive of undetected links in the transmission chain since direct transmission between patients is unlikely in the light of the existing temporal gap. These missing links that can take the form of undetected colonized patients, healthcare personnel or other environmental niches should thus pose a major concern towards infection control and prevention and, can ensure the persistence over such large temporal spans. Globally, the genomic clusters identified are mostly congruent with ST clade distribution. These demonstrate the dissemination of AMR strains across different hospital wards with the potential for the lateral acquisition of drug resistance genes as demonstrated by the similar genetic context of *bla_CTX-M-15_* across the distantly related ST15/307/348/37/70 strains.

Besides ESBL-producing strains, the emergence of KPC-producing isolates in the studied hospital unit is not associated with the pandemic spread of *K. pneumoniae* ST258 clone, which has predominantly been identified from the USA and other European countries. In Portugal, the ST258 has not been described either at the hospital level or in the community [7, 10, 49, 50]. KPC-producing isolates were distributed among different STs, of which ST147 was the predominant one and ST960 isolated for first time by Manageiro *et al.* [49]. The transposon Tn*4401* has been associated with *bla_KPC_* genes. Isoforms of Tn*4401* have been reported, differing by deletions of 68 to 255 bp in the upstream region of the *bla_KPC_* gene [43]. The Tn*4401d* isoform was initially identified by some of us in a ST11 *K. pneumoniae* clinical isolate obtained from the blood of a neonate at a different Lisbon hospital in March 2009 (data not shown) [7]. The similarity in genetic background of *bla_KPC-3_* genes supports a scenario of local emergence and dissemination of the Tn*4401d* variant in Portugal. Concomitantly with KPC-3, GES-5 carbapenemase-coding gene was detected on ST147 isolates in a new integron unit (In4885) harbouring *bla_BEL-1_* and structurally similar to the previously described In1144 integron but lacking *bla_IMP-8_*. This new integron mobilization unit is structurally compatible with a precursor structure for the In1144. Both GES-5 and BEL-1 β-lactamases are usually described in *Pseudomonas aeruginosa* while showing a high dissemination potential across other species, such as *Acinetobacter baumannii* or *Morganella morganii *, and usually within an integron mobilization unit [51–54]. Moreover, a GES-5-producing Enterobacteriaceae was first isolated in Portugal from aquatic environmental samples and, a *K. pneumoniae* carrying the *bla_GES-5_* gene was isolated in a hospital in northern Portugal [44, 55]. Taken together with these latter findings, the data herein reported support for the successful emergence and dissemination of GES-5-producing *K. pneumoniae* as a cause of healthcare-associated infection.

By providing a high-resolution genome-wide perspective on the epidemiology of ESBL and carbapenemase-producing *K. pneumoniae *, this study contributed to the adoption of appropriate intervention and prevention strategies. Infection control measures, including contact isolation, barrier precaution and rectal and pharyngeal surveillance cultures in infected patients and screening of patients, especially those receiving long-term healthcare and/or extensive antibiotic treatment, and environmental periodic screening is required for controlling and preventing transmission.

## Data Bibliography

1. Pasteur’s MLST Web site (https://bigsdb.pasteur.fr/klebsiella/klebsiella.html) September 2017.

2. MLST 1.8 (https://cge.cbs.dtu.dk/) December 2018.

3. PlasmidFinder (https://cge.cbs.dtu.dk/) January 2019.

## Supplementary Data

Supplementary material 1
