# Supplementary material for: Whole-genome sequencing resolves a polyclonal outbreak by extended-spectrum beta-lactam and carbapenem-resistant Klebsiella pneumoniae in a Portuguese tertiary-care hospital
Source: Microb Genom. 2020 Apr 1;7(6):000349. doi: 10.1099/mgen.0.000349 (PMC8627661; doi:10.1099/mgen.0.000349)
Supplement: Supplementary material 1 [file mgen-7-0349-s001.pdf]

**Supplementary Table S1** - Genetic and phenotypic characterization of the clinical isolates included in the study. Genetic features such as the sequence type (ST), capsular locus type (KL), antigen-O locus type (O-locus), plasmid replicons and detected antimicrobial resistance genes are shown for each isolate. Phenotypic drug susceptibility testing data is shown for amoxicilin-clavulanate (AMC), cefoxitin (FOX), cefotaxime (CTX), ceftazidime (CAZ), imipenem (IPM), gentamicin (GM) and ciprofloxacin (CIP).

| ID | Patient | ST | KL | O-locus | AMC | FOX | CTX | CAZ | IPM | GM | CIP | Ward[a] | Plasmid Replicons | Resistance Enzymes |
|---|---|---|---|---|---|---|---|---|---|---|---|---|---|---|
| Kp4845 | P1 | ST15 | KL112 | O1v1 | R | R | R | R | S | R | R | ICU | IncFIA(HI1), IncFIB(K), IncFII, IncFIB(pQil), IncFII(K), Col(MGD2), IncR, ColpVC, ColRNAI | CTX-M-15, TEM-1, OXA-1, SHV-106, APH(3'')-Ib, APH(6)-Id, AAC(3)-Iia, AAC(6')-Ib-cr, dfr14 |
| Kp4846 | P1 | ST15 | KL112 | O1v1 | R | R | R | R | S | R | R | ICU | IncFIA(HI1), IncFIB(K), IncFII, IncFIB(pQil), IncFII(K), Col(MGD2), IncR, ColRNAI | CTX-M-15, TEM-1, OXA-1, SHV-106, APH(3'')-Ib, APH(6)-Id, AAC(3)-Iia, AAC(6')-Ib-cr, dfr14 |
| Kp4847 | P1 | ST15 | KL112 | O1v1 | R | R | R | R | S | R | R | ICU | IncFIA(HI1), IncFIB(K), IncFII, IncFIB(pQil), IncFII(K), Col(MGD2), IncR, ColRNAI | CTX-M-15, TEM-1, OXA-1, SHV-106, APH(3'')-Ib, APH(6)-Id, AAC(3)-Iia, AAC(6')-Ib-cr, dfr14 |
| Kp4848 | P1 | ST15 | KL112 | O1v1 | R | R | R | R | S | R | R | ICU | IncFIA(HI1), IncFII, IncFIB(K), Col(MGD2), IncR, ColRNAI, IncL/M(pOXA-48) | CTX-M-15, TEM-1, OXA-1, SHV-106, APH(3'')-Ib, APH(6)-Id, AAC(3)-Iia, AAC(6')-Ib-cr, dfr14 |
| Kp4849 | P26 | ST15 | KL112 | O1v1 | R | R | R | R | S | R | R | OC | IncFIA(HI1), IncFII, IncFIB(K), Col(MGD2), IncR, ColRNAI | CTX-M-15, TEM-1, OXA-1, SHV-106, APH(3'')-Ib, APH(6)-Id, AAC(3)-Iia, AAC(6')-Ib-cr, dfr14 |
| Kp4850 | P1 | ST15 | KL112 | O1v1 | R | R | R | R | S | R | R | ICU | IncFIA(HI1), IncFIB(pQil), IncFII, IncFIB(K), IncFII(K), Col(MGD2), IncR, ColRNAI | CTX-M-15, TEM-1, OXA-1, SHV-106, APH(3'')-Ib, APH(6)-Id, AAC(3)-Iia, AAC(6')-Ib-cr, dfr14 |
| Kp4851 | P1 | ST15 | KL112 | O1v1 | R | S | R | R | S | S | R | ICU | IncFIA(HI1), IncFIB(pQil), IncFII, IncFIB(K), IncFII(K), IncR, ColRNAI | CTX-M-15, TEM-1, OXA-1, SHV-106, APH(3'')-Ib, APH(6)-Id, AAC(3)-Iia, AAC(6')-Ib-cr, dfr14 |
| Kp4852 | P2 | ST960 | KL10 | O2v2 | R | R | R | R | R | S | S | MED | IncFIA(HI1), IncFII(K), IncN, Col(MGD2), ColRNAI | KPC-3, TEM-150, OXA-1, SHV-164 , APH(3'')-Ib, APH(6)-Id, AAC(6')-Ib-cr |
| Kp4853 | P3 | ST307 | KL102 | O2v2 | R | S | R | R | S | S | R | OC | IncFIB(K), IncFII(K), Col(MGD2), ColRNAI | CTX-M-15, TEM-1, OXA-1, SHV-106, APH(3'')-Ib, APH(6)-Id, AAC(3)-Iia, AAC(6')-Ib-cr, QnrB1, dfr14, Sul2, tetA |
| Kp4854 | P3 | ST307 | KL102 | O2v2 | R | S | R | R | S | S | R | SURG | IncFIB(K), IncFII(K), Col(MGD2), ColpVC, ColRNAI | CTX-M-15, TEM-1, OXA-1, SHV-106, APH(3'')-Ib, APH(6)-Id, AAC(3)-Iia, AAC(6')-Ib-cr, QnrB1, dfr14, Sul2, tetA |
| Kp4855 | P4 | ST37 | KL15 | O4 | R | S | R | R | S | R | S | ICU | IncFIB(K), IncFII(K), IncFIB(Mar), Col(MGD2), ColRNAI | CTX-M-15, TEM-1, OXA-1, SHV-187, APH(3'')-Ib, APH(6)-Id, AAC(3)-Iia, AAC(6')-Ib-cr, QnrB1, dfr14, Sul2, tetA |
| Kp4856 | P5 | ST348 | KL62 | O1v1 | R | S | R | R | S | R | S | SURG | IncFIB(K), IncFII(K), Col(MGD2), ColRNAI | CTX-M-15, TEM-1, OXA-1, SHV-110, APH(3'')-Ib, APH(6)-Id, AAC(3)-Iia, AAC(6')-Ib-cr, QnrB1, dfr14, Sul2, tetA |
| Kp4857 | P6 | ST15 | KL112 | O1v1 | R | R | R | R | S | S | R | ICU | IncFIA(HI1), IncFIB(K), IncFII, IncFIB(pQil), IncFII(K), IncR, ColRNAI | CTX-M-15, TEM-1, OXA-1, SHV-106, APH(3'')-Ib, APH(6)-Id, AAC(3)-Iia, AAC(6')-Ib-cr, dfr14 |
| Kp4858 | P7 | ST423 | KL8 | O2v2 | R | S | R | R | S | S | S | MED | IncFIB(K), IncN, Col(MGD2), ColRNAI | CTX-M-32, SHV-187, FosA6 |
| Kp4859 | P8 | ST423 | KL8 | O2v2 | S | S | R | R | S | S | S | SURG | IncFIB(K), IncN, Col(MGD2), ColpVC, ColRNAI | CTX-M-32, SHV-187, FosA6 |
| Kp4860 | P9 | ST307 | KL102 | O2v2 | R | S | R | R | S | R | R | ICU | IncFIB(K), IncFII(K), Col(MGD2), ColRNAI | CTX-M-15, TEM-1, OXA-1, SHV-106, APH(3'')-Ib, APH(6)-Id, AAC(3)-Iia, AAC(6')-Ib-cr, QnrB1, dfr14, Sul2, tetA |
| Kp4861 | P10 | ST17 | KL25 | O5 | R | S | R | S | S | s | S | ICU | IncFII, IncI1, IncFIB(K), Col(MGD2), ColRNAI | CTX-M-8, SHV-187, aadA2, dfr21, Sul1 |
| Kp4862 | P11 | ST348 | KL62 | O1v1 | R | S | R | R | S | R | R | SURG | IncFIB(K), IncFII(K), Col(MGD2), ColRNAI | CTX-M-15, TEM-1, OXA-1, SHV-110, APH(3'')-Ib, APH(6)-Id, AAC(3)-Iia, AAC(6')-Ib-cr, QnrB1, dfr14, Sul2, tetA |
| Kp4864 | P13 | ST35 | KL22 | O1v1 | S | R | R | R | S | S | S | SURG | IncFIB(K), ColRNAI | CTX-M-32, SHV-33, tetD |
| Kp4865 | P14 | ST70 | KL136 | O1v2 | R | S | R | R | S | R | R | SURG | IncFIB(K), IncFII(K), IncR, ColpVC, ColRNAI | CTX-M-15, TEM-1, OXA-1, SHV-32, APH(3'')-Ib, APH(6)-Id, AAC(3)-Iia, AAC(6')-Ib-cr, QnrB1, dfr14, Sul2 |
| Kp4866 | P15 | 0 | KL128 | OL104 | | | | | | | | | Col(MGD2), IncR, ColRNAI | AAC(6')Ib-cr, APH(3')-Ia, ARR-3, DHA-17, OXA-1, blaOXY-2-10, catB3, mph(A), QnrB4, sul1 |
| Kp4867 | P15 | ST15 | KL112 | O1v1 | R | R | R | R | S | R | R | ICU | IncFIA(HI1), IncFII, IncFIB(K), IncFII(K), Col(MGD2), IncR, ColRNAI | CTX-M-15, TEM-1, OXA-1, SHV-106, APH(3'')-Ib, APH(6)-Id, AAC(3)-Iia, AAC(6')-Ib-cr, dfr14 |
| Kp4868 | P15 | ST15 | KL112 | O1v1 | R | R | R | R | S | R | R | ICU | IncFIA(HI1), IncFII, IncFIB(K), IncR, ColRNAI | CTX-M-15, TEM-1, OXA-1, SHV-106, APH(3'')-Ib, APH(6)-Id, AAC(3)-Iia, AAC(6')-Ib-cr, dfr14 |

| | | | | | | | | | | | | | | |
|---|---|---|---|---|---|---|---|---|---|---|---|---|---|---|
| Kp4869 | P26 | ST15 | KL112 | O1v1 | R | R | R | R | S | R | R | OC | IncFIA(HI1), IncFII, IncFIB(K), Col(MGD2), IncR, ColRNAI | CTX-M-15, TEM-1, OXA-1, SHV-106, APH(3'')-Ib, APH(6)-Id, AAC(3)-Iia, AAC(6')-Ib-cr, dfr14 |
| Kp4870 | P15 | ST15 | KL112 | O1v1 | R | R | R | R | S | R | R | ICU | IncFIA(HI1), IncFII, IncFIB(K), Col(MGD2), IncR, ColpVC, ColRNAI | CTX-M-15, TEM-1, OXA-1, SHV-106, APH(3'')-Ib, APH(6)-Id, AAC(3)-Iia, AAC(6')-Ib-cr, dfr14 |
| Kp4871 | P16 | ST307 | KL102 | O2v2 | R | R | R | R | S | R | R | MED | IncFIB(K), IncFIB(pQil), IncFII(K), Col(MGD2), ColRNAI | CTX-M-15, TEM-1, OXA-1, SHV-106, APH(3'')-Ib, APH(6)-Id, AAC(3)-Iia, AAC(6')-Ib-cr, QnrB1, dfr14, Sul2, tetA |
| Kp4872 | P17 | ST307 | KL102 | O2v2 | R | R | R | R | S | R | R | ICU | IncFIB(K), IncFIB(pQil), IncFII(K), Col(MGD2), ColRNAI | CTX-M-15, TEM-1, OXA-1, SHV-106, APH(3'')-Ib, APH(6)-Id, AAC(3)-Iia, AAC(6')-Ib-cr, QnrB1, dfr14, Sul2, tetA |
| Kp4873 | P17 | ST307 | KL102 | O2v2 | R | R | R | R | S | S | R | ICU | IncFIA(HI1), IncFIB(K), IncFIB(pQil), IncFII(K), Col(MGD2), ColRNAI | CTX-M-15, TEM-1, OXA-1, SHV-106, APH(3'')-Ib, APH(6)-Id, AAC(3)-Iia, AAC(6')-Ib-cr, QnrB1, dfr14, Sul2, tetA |
| Kp4874 | P17 | ST307 | KL102 | O2v2 | R | R | R | R | S | R | R | ICU | IncFIB(K), IncFII(K), Col(MGD2), ColRNAI | CTX-M-15, TEM-1, OXA-1, SHV-106, APH(3'')-Ib, APH(6)-Id, AAC(3)-Iia, AAC(6')-Ib-cr, QnrB1, dfr14, Sul2, tetA |
| Kp4875 | P17 | ST307 | KL102 | O2v2 | R | R | R | R | S | R | R | ICU | IncFIB(K), IncFIB(pQil), IncFII(K), Col(MGD2), ColpVC, ColRNAI | CTX-M-15, TEM-1, OXA-1, SHV-106, APH(3'')-Ib, APH(6)-Id, AAC(3)-Iia, AAC(6')-Ib-cr, QnrB1, dfr14, Sul2, tetA |
| Kp4876 | P17 | ST307 | KL102 | O2v2 | R | R | R | R | S | R | R | ICU | IncFIB(K), IncFIB(pQil), IncFII(K), Col(MGD2), ColRNAI | CTX-M-15, TEM-1, OXA-1, SHV-106, APH(3'')-Ib, APH(6)-Id, AAC(3)-Iia, AAC(6')-Ib-cr, QnrB1, dfr14, Sul2, tetA |
| Kp4877 | P18 | ST307 | KL102 | O2v2 | R | S | R | R | S | R | R | MED | IncFIB(K), IncFIB(pQil), Col(MGD2), ColpVC, ColRNAI | CTX-M-15, TEM-1, OXA-1, SHV-106, APH(3'')-Ib, APH(6)-Id, AAC(3)-Iia, AAC(6')-Ib-cr, QnrB1, dfr14, Sul2, tetA |
| Kp4878 | P19 | ST307 | KL102 | O2v2 | R | R | R | R | S | R | R | ICU | IncFIB(K), IncFIB(pQil), Col(MGD2), ColRNAI | CTX-M-15, TEM-1, OXA-1, SHV-106, APH(3'')-Ib, APH(6)-Id, AAC(3)-Iia, AAC(6')-Ib-cr, QnrB1, dfr14, Sul2, tetA |
| Kp4879 | P19 | ST307 | KL102 | O2v2 | R | R | R | R | S | R | R | ICU | IncFIB(K), IncFIB(pQil), Col(MGD2), IncR, ColRNAI | CTX-M-15, TEM-1, OXA-1, SHV-106, APH(3'')-Ib, APH(6)-Id, AAC(3)-Iia, AAC(6')-Ib-cr, QnrB1, dfr14, Sul2, tetA |
| Kp4880 | P19 | ST307 | KL102 | O2v2 | R | R | R | R | S | R | R | ICU | IncFIA(HI1), IncFIB(pQil), IncFIB(K), Col(MGD2), ColRNAI | CTX-M-15, TEM-1, OXA-1, SHV-106, APH(3'')-Ib, APH(6)-Id, AAC(3)-Iia, AAC(6')-Ib-cr, QnrB1, dfr14, Sul2, tetA |
| Kp4882 | P21 | ST147 | KL64 | O2v1 | R | R | R | R | R | S | R | ICU | IncFIA(HI1), IncFIB(K), IncFII(K), Col(MGD2), ColRNAI | KPC-3, GES-5, SHV-11, BEL-1, TEM-150, OXA-9, APH(3'')-Ib, APH(6)-Id, aadA1, AAC(6')-Ib-cr, dfr14, Sul2 |
| Kp4884 | P23 | ST147 | KL64 | O2v1 | R | R | R | R | R | R | R | ICU | IncFIA(HI1), IncFIB(K), IncFII(K), Col(MGD2), ColRNAI | KPC-3, GES-5, SHV-11, BEL-1, TEM-150, OXA-9, APH(3'')-Ib, APH(6)-Id, aadA1, AAC(6')-Ib-cr, dfr14, Sul2 |
| Kp4885 | P23 | ST147 | KL64 | O2v1 | R | R | R | R | R | R | R | ICU | IncFIA(HI1), IncFIB(K), IncFII(K), ColRNAI | KPC-3, GES-5, SHV-11, BEL-1, TEM-150, OXA-9, APH(3'')-Ib, APH(6)-Id, aadA1, AAC(6')-Ib-cr, dfr14, Sul2 |
| Kp4886 | P24 | ST147 | KL64 | O2v1 | R | R | R | R | R | R | R | OR | IncFIA(HI1), IncFIB(K), IncFII(K), Col(MGD2), ColpVC, ColRNAI | KPC-3, GES-5, SHV-11, BEL-1, TEM-150, OXA-9, APH(3'')-Ib, APH(6)-Id, aadA1, AAC(6')-Ib-cr, dfr14, Sul2 |
| Kp4887 | P25 | ST147 | KL64 | O2v1 | R | R | R | R | R | S | R | OR | IncFIA(HI1), IncFIB(K), IncFII(K), Col(MGD2), IncR, ColRNAI | KPC-3, GES-5, SHV-11, BEL-1, TEM-150, OXA-9, APH(3'')-Ib, APH(6)-Id, aadA1, AAC(6')-Ib-cr, dfr14, Sul2 |

a Hospital ward: intensive care unit (ICU), observation rooms (OR), surgery (SURG), medicine (MED) or outpatient clinic (OC).

**Supplementary Table S2** - Isolates with publicly available sequence data belonging to ST15, ST147 or ST307 and included in the study for comparison purposes

| Accession | Study Accession | Country | ST | Publication [Pubmed ID] |
|---|---|---|---|---|
| ASM216691v1 | PRJNA354908 | Colombia | ST307 | Villa L et al, Microb Genom, 2017 Apr;3(4):e000110 [PMID:28785421] |
| ASM216695v1 | PRJNA354908 | Italy | ST307 | Villa L et al, Microb Genom, 2017 Apr;3(4):e000110 [PMID:28785421] |
| ASM216696v1 | PRJNA354908 | Colombia | ST307 | Villa L et al, Microb Genom, 2017 Apr;3(4):e000110 [PMID:28785421] |
| ASM216702v1 | PRJNA354908 | United Kingdom | ST307 | Villa L et al, Microb Genom, 2017 Apr;3(4):e000110 [PMID:28785421] |
| ASM216703v1 | PRJNA354908 | United Kingdom | ST307 | Villa L et al, Microb Genom, 2017 Apr;3(4):e000110 [PMID:28785421] |
| ASM216706v1 | PRJNA354908 | United Kingdom | ST307 | Villa L et al, Microb Genom, 2017 Apr;3(4):e000110 [PMID:28785421] |
| ASM216707v1 | PRJNA354908 | United Kingdom | ST307 | Villa L et al, Microb Genom, 2017 Apr;3(4):e000110 [PMID:28785421] |
| ASM216713v1 | PRJNA354908 | United Kingdom | ST307 | Villa L et al, Microb Genom, 2017 Apr;3(4):e000110 [PMID:28785421] |
| ASM216714v1 | PRJNA354908 | United Kingdom | ST307 | Villa L et al, Microb Genom, 2017 Apr;3(4):e000110 [PMID:28785421] |
| ASM300617v1 | PRJNA419295 | Pakistan | ST147 | Avgoulea et al, Antimicrob Agents Chemother, 2018 Jun; 62(7) [PMID:29661874] |
| ASM303438v1 | PRJNA419295 | Greece | ST147 | Avgoulea et al, Antimicrob Agents Chemother, 2018 Jun; 62(7) [PMID:29661874] |
| ASM303443v1 | PRJNA419295 | Greece | ST147 | Avgoulea et al, Antimicrob Agents Chemother, 2018 Jun; 62(7) [PMID:29661874] |
| ASM303448v1 | PRJNA419295 | Greece | ST147 | Avgoulea et al, Antimicrob Agents Chemother, 2018 Jun; 62(7) [PMID:29661874] |
| ASM303456v1 | PRJNA419295 | Greece | ST147 | Avgoulea et al, Antimicrob Agents Chemother, 2018 Jun; 62(7) [PMID:29661874] |
| DRR076945 | PRJDB5317 | Vietnam | ST15 | Tada et al, BMC Infect Dis, 2017 [PMID:28676118] |
| DRR076946 | PRJDB5317 | Vietnam | ST15 | Tada et al, BMC Infect Dis, 2017 [PMID:28676118] |
| DRR076947 | PRJDB5317 | Vietnam | ST15 | Tada et al, BMC Infect Dis, 2017 [PMID:28676118] |
| DRR076948 | PRJDB5317 | Vietnam | ST15 | Tada et al, BMC Infect Dis, 2017 [PMID:28676118] |
| DRR076949 | PRJDB5317 | Vietnam | ST15 | Tada et al, BMC Infect Dis, 2017 [PMID:28676118] |
| DRR076950 | PRJDB5317 | Vietnam | ST15 | Tada et al, BMC Infect Dis, 2017 [PMID:28676118] |
| DRR076951 | PRJDB5317 | Vietnam | ST15 | Tada et al, BMC Infect Dis, 2017 [PMID:28676118] |
| DRR076952 | PRJDB5317 | Vietnam | ST15 | Tada et al, BMC Infect Dis, 2017 [PMID:28676118] |
| DRR076953 | PRJDB5317 | Vietnam | ST15 | Tada et al, BMC Infect Dis, 2017 [PMID:28676118] |
| DRR076954 | PRJDB5317 | Vietnam | ST15 | Tada et al, BMC Infect Dis, 2017 [PMID:28676118] |
| DRR076955 | PRJDB5317 | Vietnam | ST307 | Tada et al, BMC Infect Dis, 2017 [PMID:28676118] |
| DRR076956 | PRJDB5317 | Vietnam | ST15 | Tada et al, BMC Infect Dis, 2017 [PMID:28676118] |
| DRR076957 | PRJDB5317 | Vietnam | ST15 | Tada et al, BMC Infect Dis, 2017 [PMID:28676118] |
| DRR076958 | PRJDB5317 | Vietnam | ST15 | Tada et al, BMC Infect Dis, 2017 [PMID:28676118] |
| DRR076959 | PRJDB5317 | Vietnam | ST15 | Tada et al, BMC Infect Dis, 2017 [PMID:28676118] |
| DRR076961 | PRJDB5317 | Vietnam | ST15 | Tada et al, BMC Infect Dis, 2017 [PMID:28676118] |
| DRR076962 | PRJDB5317 | Vietnam | ST15 | Tada et al, BMC Infect Dis, 2017 [PMID:28676118] |
| DRR076963 | PRJDB5317 | Vietnam | ST15 | Tada et al, BMC Infect Dis, 2017 [PMID:28676118] |
| DRR076967 | PRJDB5317 | Vietnam | ST147 | Tada et al, BMC Infect Dis, 2017 [PMID:28676118] |
| DRR076969 | PRJDB5317 | Vietnam | ST15 | Tada et al, BMC Infect Dis, 2017 [PMID:28676118] |
| ERR1740512 | PRJEB18059 | Germany | ST15 | Becker et al, Front Microbiol, 2018 [PMID:29527200] |
| ERR1740513 | PRJEB18059 | Germany | ST15 | Becker et al, Front Microbiol, 2018 [PMID:29527200] |
| ERR1740519 | PRJEB18059 | Germany | ST15 | Becker et al, Front Microbiol, 2018 [PMID:29527200] |
| ERR1740521 | PRJEB18059 | Germany | ST15 | Becker et al, Front Microbiol, 2018 [PMID:29527200] |
| ERR1740522 | PRJEB18059 | Germany | ST15 | Becker et al, Front Microbiol, 2018 [PMID:29527200] |
| ERR1740524 | PRJEB18059 | Germany | ST15 | Becker et al, Front Microbiol, 2018 [PMID:29527200] |
| ERR1740526 | PRJEB18059 | Germany | ST15 | Becker et al, Front Microbiol, 2018 [PMID:29527200] |
| ERR1740534 | PRJEB18059 | Germany | ST15 | Becker et al, Front Microbiol, 2018 [PMID:29527200] |
| ERR1740535 | PRJEB18059 | Germany | ST15 | Becker et al, Front Microbiol, 2018 [PMID:29527200] |
| ERR1740536 | PRJEB18059 | Germany | ST15 | Becker et al, Front Microbiol, 2018 [PMID:29527200] |
| ERR1740537 | PRJEB18059 | Germany | ST15 | Becker et al, Front Microbiol, 2018 [PMID:29527200] |
| ERR1740539 | PRJEB18059 | Germany | ST15 | Becker et al, Front Microbiol, 2018 [PMID:29527200] |
| ERR1740540 | PRJEB18059 | Germany | ST15 | Becker et al, Front Microbiol, 2018 [PMID:29527200] |
| ERR1740541 | PRJEB18059 | Germany | ST15 | Becker et al, Front Microbiol, 2018 [PMID:29527200] |
| ERR1740542 | PRJEB18059 | Germany | ST15 | Becker et al, Front Microbiol, 2018 [PMID:29527200] |
| ERR1740543 | PRJEB18059 | Germany | ST15 | Becker et al, Front Microbiol, 2018 [PMID:29527200] |
| ERR1740544 | PRJEB18059 | Germany | ST15 | Becker et al, Front Microbiol, 2018 [PMID:29527200] |
| ERR1740546 | PRJEB18059 | Germany | ST15 | Becker et al, Front Microbiol, 2018 [PMID:29527200] |
| ERR1740548 | PRJEB18059 | Germany | ST15 | Becker et al, Front Microbiol, 2018 [PMID:29527200] |
| ERR1740549 | PRJEB18059 | Germany | ST15 | Becker et al, Front Microbiol, 2018 [PMID:29527200] |
| ERR1740550 | PRJEB18059 | Germany | ST15 | Becker et al, Front Microbiol, 2018 [PMID:29527200] |
| ERR1740551 | PRJEB18059 | Germany | ST15 | Becker et al, Front Microbiol, 2018 [PMID:29527200] |
| ERR1740553 | PRJEB18059 | Germany | ST15 | Becker et al, Front Microbiol, 2018 [PMID:29527200] |
| ERR1740554 | PRJEB18059 | Germany | ST15 | Becker et al, Front Microbiol, 2018 [PMID:29527200] |
| ERR1740555 | PRJEB18059 | Germany | ST15 | Becker et al, Front Microbiol, 2018 [PMID:29527200] |
| ERR1740556 | PRJEB18059 | Germany | ST15 | Becker et al, Front Microbiol, 2018 [PMID:29527200] |
| ERR1740557 | PRJEB18059 | Germany | ST15 | Becker et al, Front Microbiol, 2018 [PMID:29527200] |
| ERR1740558 | PRJEB18059 | Germany | ST15 | Becker et al, Front Microbiol, 2018 [PMID:29527200] |
| ERR1740559 | PRJEB18059 | Germany | ST15 | Becker et al, Front Microbiol, 2018 [PMID:29527200] |
| ERR1740560 | PRJEB18059 | Germany | ST15 | Becker et al, Front Microbiol, 2018 [PMID:29527200] |
| ERR1740562 | PRJEB18059 | Germany | ST15 | Becker et al, Front Microbiol, 2018 [PMID:29527200] |
| ERR1740563 | PRJEB18059 | Germany | ST15 | Becker et al, Front Microbiol, 2018 [PMID:29527200] |

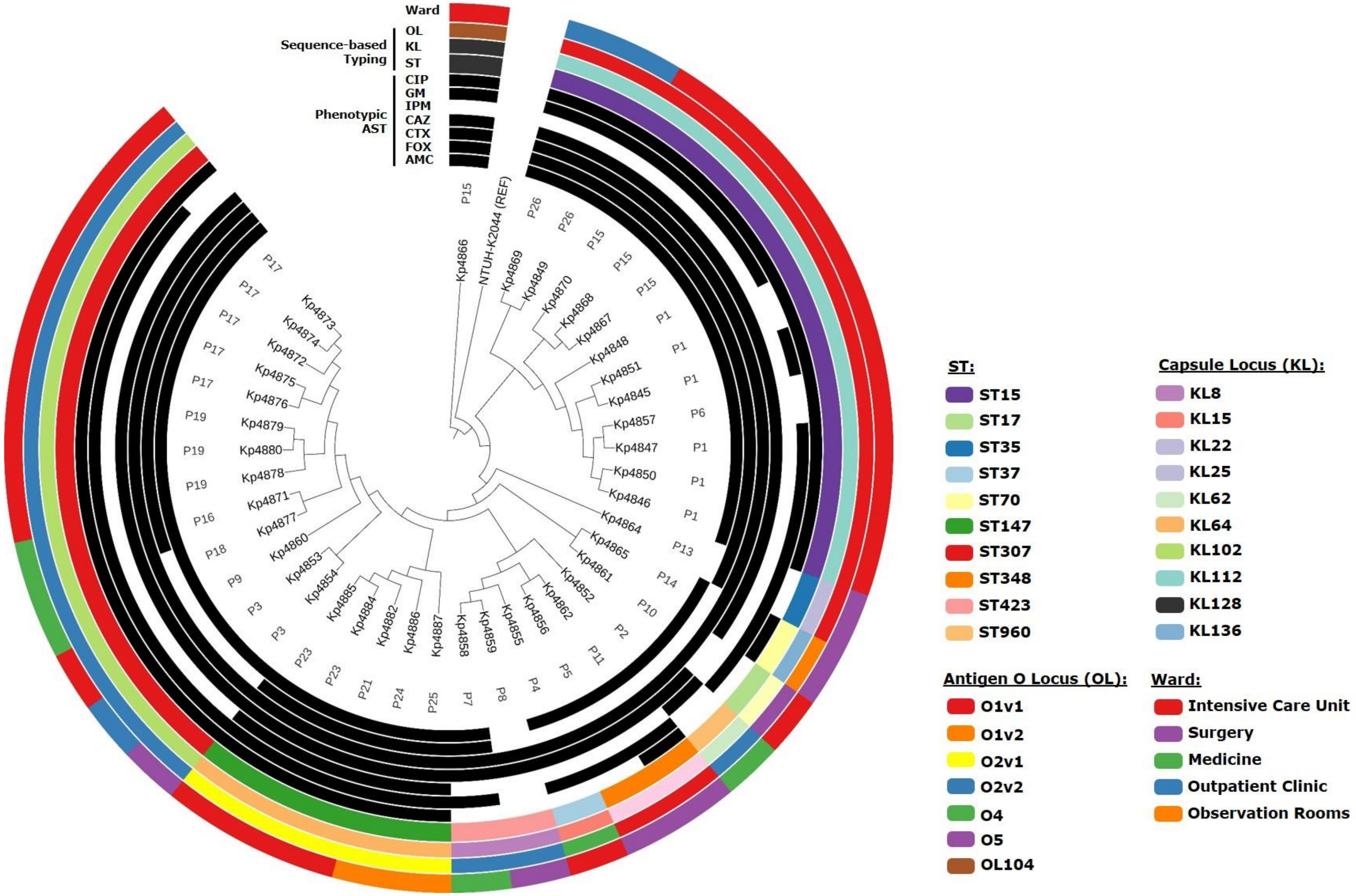


**Supplementary Figure S1** - Maximum-likelihood phylogenetic tree based on 59 968 variant SNPs for the 39 clinical isolates included in the study. The tree was rooted with *Klebsiella oxytoca* Kp4866 sequenced in the study. The tree is shown as a cladogram (see **Figure 1** for the phylogram) and annotated with phenotypic drug susceptibility data to amoxicillin-clavulanate (AMC), cefoxitin (FOX), cefotaxime (CTX), ceftazidime (CAZ), imipenem (IPM), gentamicin (GM) and ciprofloxacin (CIP) where black rectangles indicate phenotypic resistance. Isolate ST, capsular locus type (KL), antigen O locus type (OL) and ward of isolation are also annotated on the tree according to the color-coded legends.

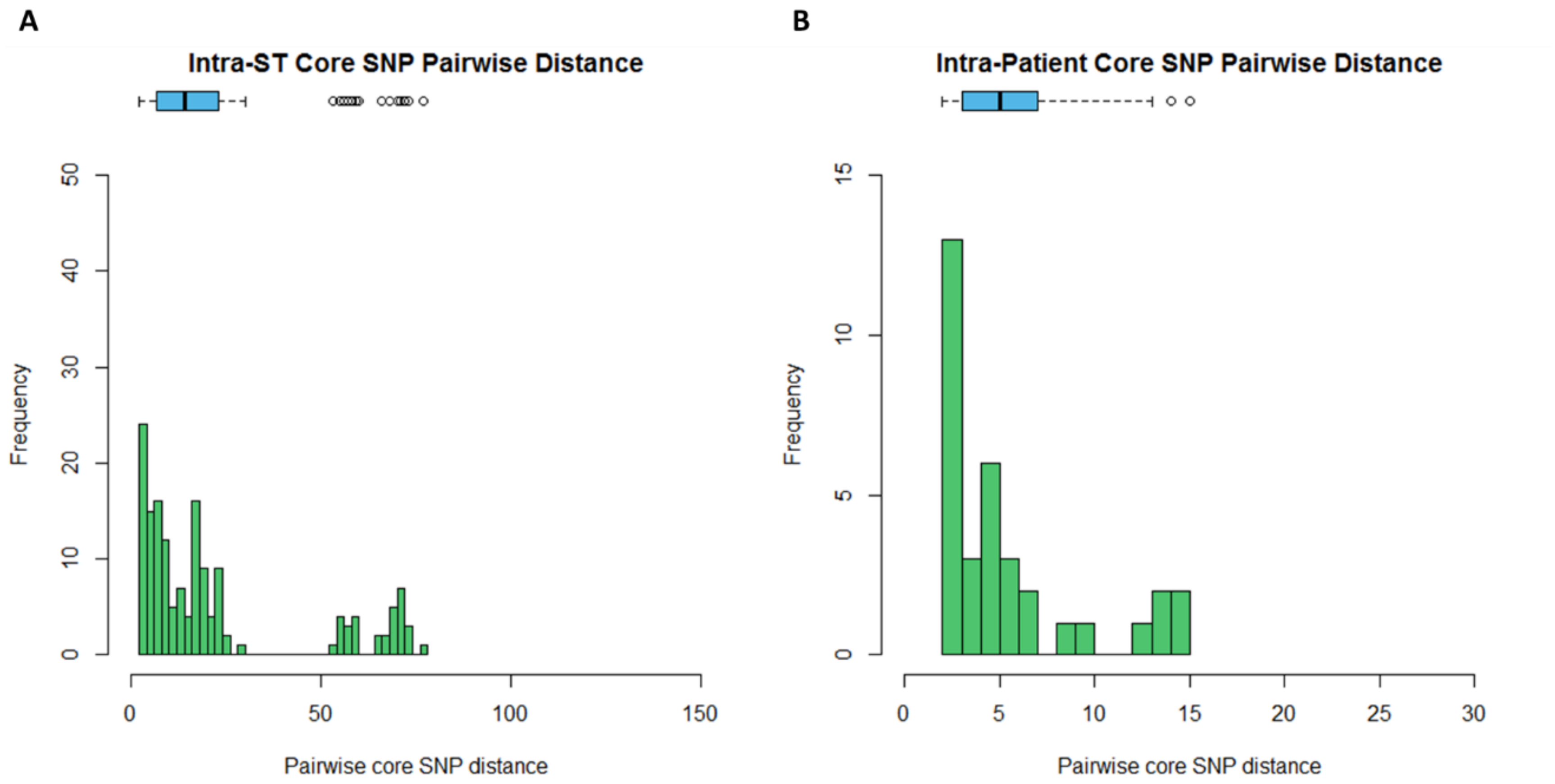


**Supplementary Figure S2** – Intra-ST (A) and Intra-Patient (B) core SNP pairwise distances showing a tighter within-patient SNP distance distribution when compared to clinical isolates within the same ST suggesting either polyclonal ST composition or ongoing dissemination in the studied setting.

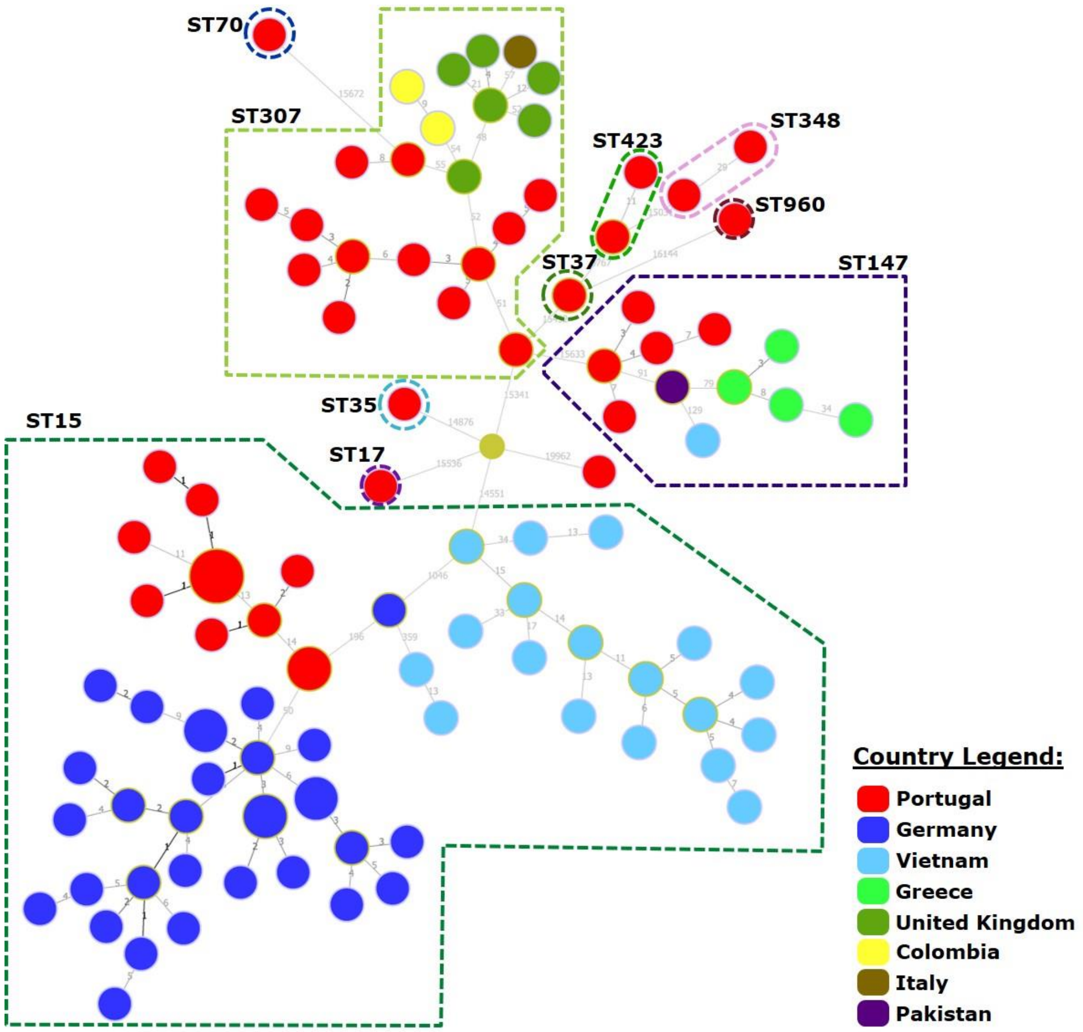


**Supplementary Figure S3** – goeBURST Full MST of all 39 *Klebsiella pneumoniae* clinical isolates included in the study along with the *Klebsiella oxytoca* Kp4866 and an additional 64 clinical isolates with publicly available sequence data, belonging to the three most prevalent STs found in the study (ST15, ST147 and ST307), and representative of external *K. pneumoniae* outbreaks in different countries worldwide (see **Supplementary Table S2**). Node coloring represents the country of origin of the isolate(s) present at each node in the tree and STs are identified in the tree. Across this dataset, the topological structure of the tree shows a clear country-wise clustering trend within each ST. Nevertheless, two ST307 nodes representing isolates from the present study are clearly split from the remaining ST307 isolates by a ST307 node from the United Kingdom. Numbers at each branch indicate the distance between nodes in SNPs; links with shorter distances are colored in black and longer distances in grayscale.
